# Sensitive and rapid quantification of C-reactive protein using quantum dot-labeled microplate immunoassay

**DOI:** 10.1186/1479-5876-10-24

**Published:** 2012-02-06

**Authors:** Yang Luo, Bo Zhang, Ming Chen, Tianlun Jiang, Daiyang Zhou, Junfu Huang, Weiling Fu

**Affiliations:** 1 Department of Laboratory Medicine, Southwest Hospital, The Third Military Medical University, Chong Qing 400038, Peoples' Republic of China; 2 Department of Laboratory Medicine, Daping Hospital, The Third Military Medical University, Chong Qing 400040, Peoples' Republic of China; 3 Department of Transfusion Medicine, Southwest Hospital, The Third Military Medical University, Chong Qing 400038, Peoples' Republic of China

**Keywords:** Cardiovascular disease, C-reactive protein, Fluoroimmunoassay, Quantification, Quantum dots

## Abstract

**Background:**

High-sensitivity C-reactive protein (hs-CRP) assay is of great clinical importance in predicting risks associated with coronary heart disease. Existing hs-CRP assays either require complex operation or have low throughput and cannot be routinely implemented in rural settings due to limited laboratory resources.

**Methods:**

We developed a novel hs-CRP assay capable of simultaneously quantifying over 90 clinical samples by using quantum dots-labeled immunoassay within a standard 96-well microplate. The specificity of the assay was enhanced by adopting two monoclonal antibodies (mAbs) that target distinct hs-CRP epitopes, serving as the coating antibody and the detection antibody, respectively. In the presence of hs-CRP antigen, the fluorescence intensity of the mAb-Ag-mAb sandwich complex captured on the microplate can be read out using a microplate reader.

**Results:**

The proposed hs-CRP assay provides a wide analytical range of 0.001-100 mg/L with a detection limit of 0.06 (0.19) μg/L within 1.5 h. The accuracy of the proposed assay has been confirmed for low coefficient of variations (CVs), 2.27% (intra-assay) and 8.52% (inter-assay), together with recoveries of 96.7-104.2%. Bland-Altman plots of 104 clinical samples exhibited good consistency among the proposed assay, commercial high-sensitivity ELISA, and nephelometry, indicating the prospects of the newly developed hs-CRP assay as an alternative to existing hs-CRP assays.

**Conclusion:**

The developed assay meets the needs of the rapid, sensitive and high-throughput determination of hs-CRP levels within a short time using minimal resources. In addition, the developed assay can also be used to detect and quantify other diagnostic biomarkers by immobilizing specific monoclonal antibodies.

## Background

Serum high-sensitivity C-reactive protein (hs-CRP) is of clinical importance as an independent marker for coronary heart disease, with stratifications corresponding to low (< 1 mg/L), moderate (1-3 mg/L), and high (> 3 mg/L) levels of cardiovascular risks [1-3] and insulin resistance [4,5].

Over the last decades, various hs-CRP assays have been developed, including the enzyme-linked immunosorbent assay (ELISA) [6,7], immunoturbidimetry [8], time-resolved immunofluorimetric assay [9], and mass spectrometry [10]. Among the assays, mass spectrometry-based measurements are the most accurate and have been widely used in proteomics [11]. Immunoturbidimetry assays and time-resolved immunofluorimetric assays provide excellent sensitivity (better than 2.8 μg/L) with a detection time of < 1 h [9]. However, these techniques require relatively expensive instrumentation, rigid operational protocols and an inflexible set of reagents. Although ELISAs are frequently performed in most clinical laboratories due to low cost and simplicity, the accuracies of traditional ELISAs vary considerably when the sample concentrations are lower than 1 μg/L. Although high-sensitivity ELISAs (hs-ELISAs) improve the detection sensitivity as low as 0.014 μg/L by signal amplification [12-14], the considerably complicated operation protocols limit their wide clinical applications [15]. Fluorescence or chemiluminescence-based techniques are useful alternatives in clinical diagnosis because of their high sensitivities, allowing them to detect small amounts of proteins and cytokines [16]. Quantum dots (QDs) are ideal dyes with excellent fluorescence intensity and photobleaching resistance ability and have been utilized for the detection of various biological analytes, such as nucleic acids [17-20], proteins [21], cells [22-24], and organic molecules [25]. Recently, Zhu et al. established an hs-CRP assay with QD labeling and immunoaffinity separation [26]. However, this assay is restricted in large-scale clinical sample determination due to its complex operation and stringent reagent requirements. In contrast, hs-CRP assays, with the ability to sensitively and economically analyze 50-100 samples simultaneously, meet the needs for the rapid and early diagnosis of heart disease. Unfortunately, no such high-throughput QDs-based hs-CRP assays have been reported until now.

In this paper, we report a low cost hs-CRP assay capable of analyzing 90 clinical serum samples simultaneously. This quantum dot-labeled microplate immunoassay (QL-MI) combined the features of QD labeling in microplate format, allowing the fluorescence intensity to be read out using a microplate reader, which is available in most clinical laboratories. In a typical QL-MI assay, two monoclonal antibodies (mAbs) against distinct epitopes of hs-CRP were applied to enhance the specificity of the assay. The hs-CRP antigen is captured by the mAb immobilized on the microplate, then the QD565 labeled detection antibody is introduced to form an mAb-Ag-mAb sandwich complex. The induced fluorescence intensity (FI) is proportional to the concentration of serum hs-CRP. In this study, the optimal operation conditions, including mAb coating concentration and incubation time, are discussed. Furthermore, two formats are designed for different clinical applications. A one-step QL-MI that requires shorter detection time can be applied for large-scale sample screening. Alternatively, a two-step QL-MI, which introduces the QD565 and mAb into the microwells separately, provides improved analytical characteristics and can be implemented in routine clinical applications. Methodological evaluation of 104 clinical serum samples confirmed that the proposed hs-CRP assay is highly comparable with existing hs-ELISAs and nephelometry.

## Methods

### Materials and reagents

All serum samples were collected from the Southwest Hospital (China) between May 2009 and Jan 2010. There were a total of 104 serum samples used in the study; 68 samples were from healthy individuals undergoing a physical health examination, and 36 samples were from patients with various inflammation conditions. All participants provided signed informed consents, and the study was approved by the Ethics Boards of the Third Military Medical University. In this study, 8 mL of peripheral blood was drawn in the morning after an overnight fast. All of the serum samples were isolated after centrifugation at 3000 rpm for 15 min and then immediately stored at -20°C; repeated freeze-thaw cycles were avoided. Prior to the assay, the frozen samples were brought to room temperature slowly and gently mixed. Next, each sample suspension was divided into four equal portions for analysis by nephelometry, hs-ELISA, one-step QL-MI, and two-step QL-MI.

High-purity CRP from human serum was obtained from EMD Biosciences (La Jolla, CA). CRP control serum was purchased from Beckman Coulter (Brea, CA). The CRP primary monoclonal antibody (clone 3) was obtained from Meridian Life Science (Saco, ME). Anti-CRP (clone 7) was obtained from Santa Cruz (San Francisco, CA). The Human CRP ELISA Kit was obtained from Phoenix Pharmaceutical (Burlingame, CA). Purified human albumin, immunoglobulin G, and hemoglobin were purchased from Pierce (Rockford, IL). The Qdot 565 ITK Streptavidin Conjugate Kit was obtained from Invitrogen (Gaithersburg, MD). Phosphate buffered saline (PBS), bovine serum albumin (BSA), sodium bicarbonate, and other reagents were obtained from Sigma-Aldrich (St. Louis, MO). Ultra-pure water (18.2 MΩ·cm) from Milli-Q (Billerica, MA) was used for the preparation of all solutions and for the cleaning of substrates. A Synergy HT Hybrid Multi-Mode Microplate Reader was obtained from BioTek (Winooski, VT) for fluorescence measurements. UV-transparent, clear-bottom microplates (96-well) were obtained from Corning (Lowell, MA). A Microplate Genie mixer from Scientific Industries (Bohemia, NY) was used for reagent mixing.

### Coating of 96-well microplates with the primary antibody

Primary CRP mAb coating was achieved through passive adsorption of the antibody onto the bottom of the microplate, according to the protocol provided by Thermo Scientific with slight modifications. Briefly, the primary monoclonal antibody was diluted in a carbonate-bicarbonate buffer (0.2 M sodium carbonate/bicarbonate, pH 9.4), and 100 μL of appropriately diluted antibody was added into each well of a 96-well microplate. The plates were covered with parafilm and incubated for 12 h at 4°C. The incubation was followed by three washes with a wash buffer (0.1 M PBS, pH 7.2, 0.15 M NaCl, with 0.05% Tween-20). Next, 300 μL of blocking buffer (wash buffer, pH 7.2, with 2% BSA) was applied to each well for 2 h to ensure that all the remaining and available binding surfaces of the plastic wells were covered. Then, the microplates were dried and stored at 4°C until use.

### Preparation of the standards and biotinylated anti-CRP

High-purity CRP was diluted in a dilution buffer (PBS, pH 7.4, with 0.05% Tween-20) from an original concentration of 500 mg/L to concentrations of 0.001, 0.01, 0.1, 1, 10, and 100 mg/L for use as calibration standards. The preparation of QD-streptavidin conjugates followed the procedure supplied by Invitrogen. Briefly, QD-streptavidin conjugates were centrifuged at 5000 rpm for 3 min prior to use. Next, 2 μL of the stock supernatant (approximately 1 μM) was immediately added to 100 μL of the secondary incubation buffer to produce a 20 nM QD-streptavidin conjugate solution. The protocol of anti-CRP biotinylation was supplied by Santa Cruz. Briefly, 10 mg of biotin was dissolved in 1 mL of anhydrous DMSO immediately before use. Biotin was then mixed with secondary CRP mAb at a concentration ratio of 80 μg/mg (biotin: mAb). After incubation at room temperature for 4 h, the unreacted biotin was removed, and the conjugated antibody was transferred into the storage buffer by gel filtration or dialysis. The QD-streptavidin conjugate or biotinylated CRP mAb was diluted using a 20% standard diluent buffer prior to use.

### Preparation of QD-antibody complex

The QD-mAb complex was prepared according to the procedure supplied by Invitrogen. Briefly, the monoclonal antibody and the QD-streptavidin conjugate were mixed and incubated with slight stirring at 25°C for 2 h to form the QD-antibody complex. Next, the QD-mAb complex was concentrated and purified prior to collecting the absorbance and fluorescence spectra for the effluent. Samples with an absorbance at 280 nm and no fluorescence at the emission maximum of the QDs contained excess antibodies. A Bradford assay was performed to calculate the coupling efficiency by determining the amount of uncoupled antibody. The conjugated antibody concentration was determined by subtracting the amount of uncoupled antibody from the total number of antibodies used in the assay. The induced concentrations of the QD-antibody conjugate were determined accordingly.

### Measurement procedures

In each well of a 96-well plate, 100 μL of the solution (serum sample or diluted hs-CRP standard) was incubated with the primary mAb that was preabsorbed onto the well at 25°C for 20 min. Each well was then washed three times with wash buffer (PBS buffer, pH 7.4, with 0.05% Tween-20) at 2 min per wash. It is essential in these experiments to place the patient samples and calibration standards on the plates in a manner that minimizes biased readings to obtain reliable results. Therefore, these measurements were performed in duplicates, and the duplicated samples were placed in different positions on the plate.

**Figure 1:**
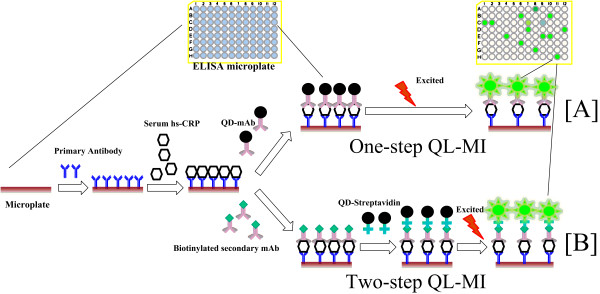
**Scheme for the two formats of QL-MI**. These assays share the same procedure at the first two steps, including monoclonal antibody coating and antigen introduction. The difference is that **A**), the QD-mAb conjugate was introduced into the microplate prior to fluorescence detection in the one-step format while **B**), the QD-streptavidin was added 20 min later than the biotinylated secondary antibody in two-step format.

For subsequent hs-CRP detection, two assay formats were applied (Figure 1): A) a one-step detection with 50 μL of the QD-mAb complex per well at a final concentration of 20 nM (Figure 1A) or B) and a two-step format that added 50 μL of biotinylated anti-CRP antibody first in each well, followed by three washes and incubation with 50 μL/well of QD-streptavidin (Figure 1B). For both assay formats, the microplate was washed three times with wash buffer to remove any unreacted QD-streptavidin complex or QD-mAb complex prior to fluorescence measurements.

### Optimization of reaction conditions

To obtain the optimum conditions for our hs-CRP assays, various analytical conditions, including the coated primary monoclonal antibody concentration (1-10 mg/L) and the concentrations of the QD-conjugated secondary mAb against hs-CRP (0.1-1 mg L^-1^), were optimized to achieve an ideal regression coefficient for the standard curve and a high signal-to-noise ratio of fluorescence intensity.

### Methodology comparison and clinical sample detection

The operational characteristics, including the precision, specificity, analytical sensitivity, and detection limits, were evaluated to validate the proposed immunoassay. All 104 clinical samples were analyzed for hs-CRP levels using one-step QL-MI, two-step QL-MI, hs-ELISA, and nephelometry (Beckman, Brea CA). Nephelometry and hs-ELISA assays were performed according to the protocols provided by the suppliers. Bland-Altman plots were performed with MedCalc (version 11.3) to evaluate the residuals plots of the proposed and reference methods. A statistically significant difference between the fluorescence intensity induced by various interferents was tested with t-tests. The results were considered statistically significant if the *P*-value was < 0.05. The calibration curves were plotted using Origin (version 8.1).

## Results and discussion

The emergence of hs-CRP as a predicting marker of future cardiovascular events calls for the rapid, low cost and high-throughput hs-CRP assays that can determine slight increases in hs-CRP levels above the normal level using readily available clinical settings. This study is aimed to establish such a high-throughput hs-CRP assay that could be implemented in clinical laboratories equipped with microplate readers. To generate a high-throughput model, the widely used 96-well microplate was adopted as the substrate to immobilize the capture antibody, a primary mAb against an epitope of hs-CRP (Figure 1). After the hs-CRP antigen was captured onto the microplate, a secondary mAb against a different epitope of the hs-CRP was added into the microwell to form a mAb-Ag-mAb complex. The adoption of two high-affinity mAbs, targeting distinct epitopes of hs-CRP, potentially eliminates the false-positive signals encountered in traditional ELISAs. According to differences in procedure of introducing the reporter (QD565), we developed the QL-MI assay in two formats: one-step QL-MI and two-step QL-MI.

**Figure 2:**
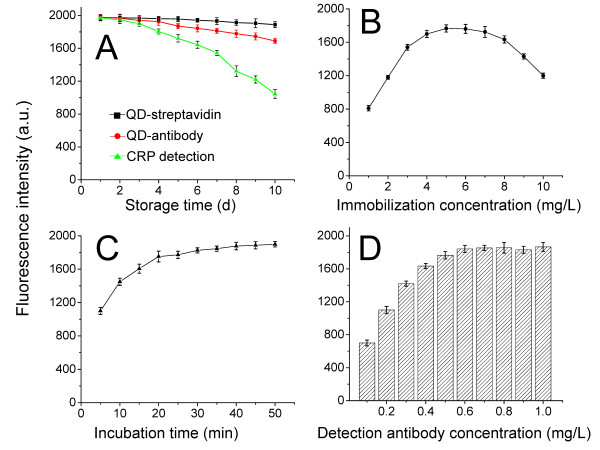
**Influence of storage time (A), immobilization concentration (B), incubation time (C), and detection antibody concentration (D) on the fluorescence intensity. **(A) Fluorescence intensity after different storage times (1-10 d) for the QD-streptavidin stock solution only (black line), QD-mAb stock solution only (red line), and 10 mg/L of the purified CRP standard using QD-mAb (green line). (**B**) Various coating concentrations (1-10 mg/L) of primary anti-CRP monoclonal antibody were employed, and 10 mg/L of serum hs-CRP was introduced into the detection well to judge the highest fluorescence intensity. (**C**) In each determination, the serum hs-CRP (10 mg/L) was incubated with 4 mg/L of coating antibody for different time periods (varied from 5-50 min with a 5-min interval) to determine the optimal reaction time. (**D**) Various coating concentrations (0.1-1 mg/L) of capture antibody were introduced into the microplate (4 mg/L coating antibody) and reacted with 10 mg/L serum CRP. Error bars indicate the standard errors of 3 independent experiments.

In the one-step assay (Figure 1A), streptavidin-coated QD565 were preconjugated with the secondary hs-CRP mAbs with a high coupling efficiency (approximately 95%). To investigate the stability of preconjugated QD-antibody complex, the storage time of the complex was studied. No obvious aggregation was observed within three days when stored at 4°C. However, aggregation can be observed in the solution when the storage time increased. Sonication or vortexing resulted in a decreased limit of detection, indicating that the resulting QD-antibody conjugate was not stable for extended storage periods. We postulate the decreased detection limit is due to the aggregation of the functionalized QDs after gradually losing their colloidal stability in the PBS or the fluorescence quenching effect by conjugated biomolecules or the remaining chemicals in the solution [27]. We further compared the extent by which the FI reduced over time for CRP detection, QD-streptavidin and QD-mAb stock solution. The results show that the QD-streptavidin stock solution has almost no obvious FI decrease within 10 days, and the FI for QD-antibody stock solution decreases ~16% of its initial value by day 10 (Figure 2A). However, in case of CRP detection, the corresponding FI decreased ~20% after 5 days of storage and ~45% after 10 days of QD-mAb storage. These results indicate that in addition to the aggregation of the QDs, inactivation of the antibody may also contribute to the decrease in fluorescence intensity observed over extend storage time. Two reasons may attribute to the decrease in fluorescence: (i) the dissociation of the antibody from the QDs-antibody conjugate and (ii) improper storage conditions (antibodies should be stored below -20°C or -80°C to avoid the inactivation of the epitopes, whereas QDs should be kept at 4°C to avoid decrease in fluorescence). For improved results, the fluorescence of the Ab-Ag-Ab complex should be measured immediately, and the longest permissible storage time should be shorter than one week in PBS buffer (pH 7.4). This shortcoming greatly limits the application of the one-step assay in routine clinical detection, although it can simplify the procedure and reduce analysis time.

To avoid the degradation caused by the long-term storage of the QD-mAb complex, a two-step format was developed. The biotinylated secondary mAb (detection antibody) was introduced into the microplate separately to react with the captured hs-CRP antigen (Figure 1B). After incubating for 20 min at 37°C, any excess or unbound detection antibodies were washed away by three rinses with wash buffer. Next, the streptavidin coated QD565 was added into the microwell followed by a 10-min incubation to allow the completion of streptavidin-biotin reaction. Lastly, the fluorescence intensity was read out using a microplate reader after washing away any unbound QD-streptavidin. Although the detection time increased by approximately 20 min due to the extra incubation and washing steps, the accuracy and sensitivity of this format are enhanced by eliminating the fluorescence decrease caused by the long-term storage of the QD-mAb complex.

### Optimization of analytical parameters

Various assay parameters (Figure 2B-D) were optimized for the hs-CRP assay including the concentration of the captured antibody, the incubation time of the hs-CRP antigen, and the concentration of the tagged detection antibody. To simplify the experiment, all our optimizations were performed using the two-step format. Figure 2B shows that the FI increases proportionally with the coating antibody concentration for concentrations lower than 4 mg/L. The FI reaches a maximum at a coating concentration of 4-6 mg/L and then decreases as the coating concentration becomes higher than 6 mg/L. This behavior is primarily attributed to the steric hindrance that prevents the formation of the Ag-Ab complex between the mAb coating and the hs-CRP antigen when the primary mAb is over-coated. Accordingly, the optimal coating mAb concentration is defined to be 4 mg/L.

Similarly, a time-dependent fluorescence intensity increase was observed when the serum incubation time increased from 5 to 30 min (Figure 2C). According to the combination kinetics between the antigen and the antibody, the FI reached a maximum at 20 min when a dynamic balance was reached between the coating mAb and the hs-CRP antigen. This balance is confirmed by the slight increase in fluorescence intensity even with a longer incubation time. Thus, 20 min was ascertained as the optimal serum incubation time. Figure 2D reveals that the optimal detection antibody (biotinylated anti-CRP) concentration is 0.6 mg/L. To evaluate the optimal washing procedure, longer incubation times with the wash buffer and higher Tween-20 concentrations were tested. The results showed that none of these changes improved the coefficients of variations (CVs) or eliminated the background measurements. The exception was that the introduction of Tween-20 in the wash buffer produced lower background fluorescence signals than the PBS buffer without Tween-20 (data not shown).

### Calibration curves and linearity of analytical results

**Figure 3:**
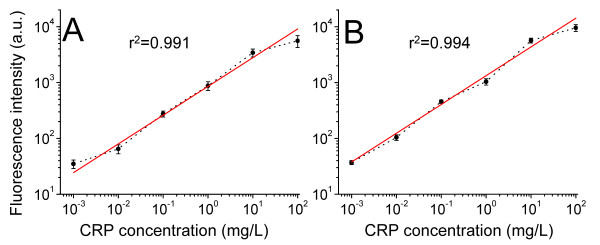
**Calibration curves for hs-CRP detection**; 0.001, 0.01, 0.1, 1, 10, and 100 mg/L standard CRP was diluted from CRP stock solution and reacted with optimal capture antibody (4 mg/L) to perform this calibration. A) The best fit for the calibration curve is y = 0.51× + 2.63 with r ^2 ^= 0.991 in the one-step format. The black solid round is the fluorescence intensities of individual hs-CRP concentrations and the red straight line is the fitted curve. B) The best fit for the calibration curve is y = 0.49× + 2.82 with r^2 ^= 0.994 in the two-step format. The black solid square represents the fluorescence intensities of individual hs-CRP concentrations with the red straight line representing the fitted curve. Error bars indicate the standard errors of 3 independent experiments.

Figure 3 shows that the proposed hs-CRP assay had a linearity of 0.001-100 mg/L, and no significant difference was observed in the linearity between the two-step and one-step formats. In the one-step assay, the regression equation had a slope of 0.51 (SD, 0.03) and a y-intercept of 2.93 (SD, 0.04) mg/L with an r^2 ^value of 0.991. Similarly, in the two-step format, the regression equation had a slope of 0.49 (SD, 0.02) and a y-intercept of 3.12 (SD, 0.03) mg/L with an r^2 ^value of 0.994. No significant difference was observed between the two analytical formats of QL-MI assay according to the r^2 ^value. For both analytical formats, the fluorescence intensity could not be accurately determined at protein concentrations > 100 mg/L due to the lack of curve linearity. To obtain accurate results, serum samples with concentrations higher than 100 mg/L should be diluted appropriately (10-fold or more) prior to detection.

### Analytical sensitivities and detection limits

The limit of detection (signal-to-noise ratio > 3) was calculated by measuring the negative control for 20 consecutive duplicates. The limit of quantification was determined by serially diluting stock solutions with calibrated dilute buffer (pH 7.4). Five replicates were measured for the dilutions, and the final diluted concentration, which had a CV < 20% between replicates, was considered the limit of quantification. The results showed that the proposed hs-CRP assay had a limit of detection of 0.06 μg/L and a limit of quantification of 0.19 μg/L (CV < 8%). Thus, the sensitivity of the assay developed in this experiment is higher than that of traditional ELISAs or other chemiluminescence assays [28] and is comparable to that of hs-ELISAs [13,29] or previously reported QD-based immunoassays [26]. For the two-step format assay, a detection limit of 0.21 μg/L and a quantification limit of 0.65 μg/L (CV < 6%) were obtained. The difference in sensitivity between the two-step and one-step formats is derived from the slight degradation of the preconjugated QD-mAb complex with time.

### Imprecision and accuracy

Duplicate tests were performed detecting very low (0.03 mg/L), low (0.3 mg/L), medium (3 mg/L), and high concentrations (30 mg/L) of CRP in quality control samples. For every sample, the tests were repeated 20 times within 1 day for intra-assay analysis and on 20 consecutive days in the same manner (mean of 3 duplicates per day) for inter-assay analysis. The mean intra-assay and inter-assay CVs were 2.27% and 9.52% for the one-step format, respectively, together with 1.75% and 2.17% for the two-step format, respectively. Our data showed that the intra-assay results in the two assay formats were almost identical, whereas the inter-assay variability of the one-step format was much higher than that of the two-step format. This is perhaps due to the significant fluorescence variability of the QD-mAb complex after long-term storage in the one-step assay, whereas the QD-streptavidin conjugate can retain highly stable fluorescence properties even when stored for a longer period of time in case of the two-step assay.

Recovery measurements were performed to assess the overall accuracy of the QD-based assay. The serum samples were spiked with a known amount of standard hs-CRP for analysis, and standard hs-CRP concentrations covered the low, medium, and high-risk patient levels. Table 1 shows that all of the recovery rates were within the range of 96.7-104.2%, and no significant differences in fluorescence intensity were observed between the two assays (*p *> 0.01).

**Table 1:** Recoveries of QL-MI (n = 20)

Original sample conc. (mg/L)	Added conc. (mg/L)	Recovered conc. (mg/L)		Recovery (%)	
		
		one-step format	two-step format	one-step format	two-step format
	0.91(0.01)^*a*^	0.89(0.05)	0.87(0.04)	98.92(1.34)	96.73(1.27)
6.32(0.07)	5.01(0.09)	5.21(0.07)	4.89(0.06)	104.21 (2.11)	97.81(2.48)
	20.31(0.15)	20.82(0.18)	20.03(0.13)	102.62(2.14)	98.72(3.06)
	150.01 (2.25)	154.23 (4.12)^*b*^	146.64(2.13)^*b*^	102.82(3.21)	97.81(2.76)

^a^Numbers in parentheses denote the standard error of 20 replicated tests

^b^These samples were diluted 10-fold before analysis

### Specificities of the QL-MI

**Figure 4:**
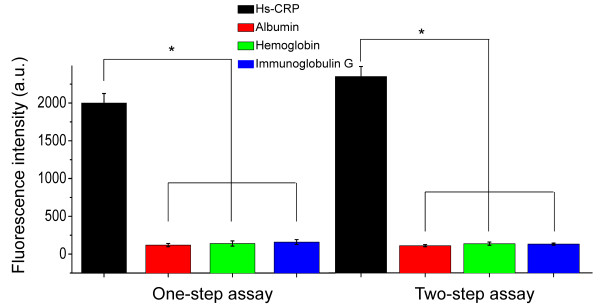
**Cross-reaction of the two assay formats**; 100 μL of a 10 mg/L solution of albumin (red column), hemoglobin (green column), and immunoglobulin G (blue column) were introduced into the coating antibody-immobilized microplate to perform the cross-reaction. No significant differences were observed between the fluorescence intensities induced by albumin, hemoglobin, and immunoglobulin G. However, the fluorescence intensities induced by nonspecific binding were significantly lower than those induced by the specific hs-CRP antigen in the one-step assay (*p *< 0.05) and in the two-step assay (*p *< 0.05). Error bars indicate the standard errors of 3 independent experiments.

Cross-reaction tests were performed by adding 100 μL of 10 mg/L interferent (albumin, immunoglobulin G, and hemoglobin) to the detection wells. The results showed that the fluorescence intensity induced by a specific Ag-Ab combination (1800 a.u. for 10 mg/L of CRP serum) was significantly stronger than that (average of ~5.6 a.u. with the range of 2.2-9.8 a.u.) from a nonspecific interaction (*p *< 0.05). These weak, nonspecific signals may be attributed to the background fluorescence interference caused by the autofluorescence of specific biomolecules in the serum matrix, which can be eliminated by introducing a reagent blank control consisting of the serum matrix without any hs-CRP. These results indicate that the proposed hs-CRP assay provides extremely high specificities, and no significant difference was observed between the two-step and one-step assays (*p *< 0.05) (Figure 4).

### Clinical sample detection and comparison

**Figure 5:**
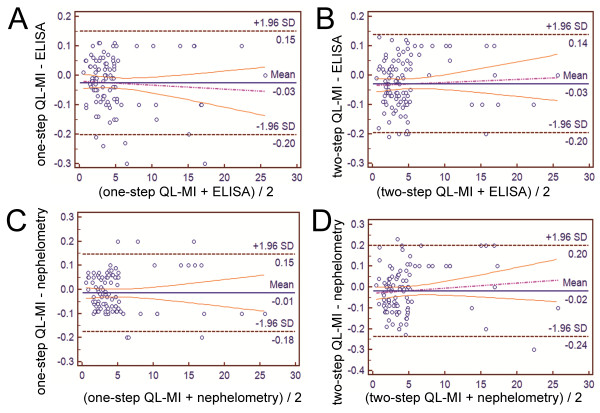
**Comparison of results for 104 clinical samples from the proposed QL-MI immunoassay, ELISA, and nephelometry**. Each sample was detected for three duplicates, and the mean of three results was taken for comparison. Black circles represent the comparison of 104 clinical samples between the one-step QL-MI and hs-ELISA (**A**); two-step QL-MI and hs-ELISA (**B**); one-step QL-MI and nephelometry (**C**); and two-step QL-MI and nephelometry (**D**). The black solid line represents the mean difference; dashed lines represent mean difference ± 1.96 times of the SD of the differences.

For methodology evaluation, 104 clinical serum samples were analyzed by ELISA, nephelometry, and the two proposed QL-MI assays. The precision results obtained from our proposed QL-MI assay are better than those of many automated hs-CRP methods [30-32]. We used the Bland-Altman difference plot analysis to compare the four diagnostic strategies: one-step QL-MI with hs-ELISA (Figure 5A), two-step QL-MI with hs-ELISA (Figure 5B), one-step QL-MI with nephelometry (Figure 5C), and two-step QL-MI with nephelometry (Figure 5D).

Compared with hs-ELISA, the Bland-Altman difference plot of the one-step QL-MI showed a mean (SD) difference (QL-MI minus hs-ELISA) of 25.48 (11.3) μg/L, and the range for the limits of agreement (d-1.96 S to d + 1.96 S, -201.2 to 150.2 μg/L) was sufficiently narrow. The two-step QL-MI provided a similar difference of 29.04 (8.5) μg/L, and the agreement limits (-196.9 to 133.8 μg/L) were sufficiently narrow. Similarly, the one-step QL-MI provided a difference of 14.71 (8.2) μg/L and a narrow range of limits (-175.6 to 146.1 μg/L) when compared with nephelometry. The two-step QL-MI provided a slight difference of 18.27 (7.4) μg/L and a narrow range of limits (-236.4 to 199.9 μg/L) when compared with nephelometry. These data suggest good consistency and clinical equivalence between the proposed hs-CRP assay and hs-ELISA or nephelometry. Table 2 summarizes a brief comparison of the advantages and disadvantages among these four assays. The hs-ELISA assay provides the best sensitivity among these four assays [12,13], whereas the nephelometry provides the shortest analytical time [33]. Compared with these two methods, our proposed QL-MI assays provide satisfactory analytical characteristics with a similar sensitivity to hs-ELISA and a shorter turnaround time.

**Table 2:** Comparison of proposed QL-MI assay with hs-ELISA and nephelometry

	QL-MI (One-step format)	QL-MI (Two-step format)	**hs-ELISA**[12,13]	**Nephelometry**^**a**^
Sensitivity	0.21 μg/L	0.06 μg/L	0.039 μg/L	1000 μg/L
Intra-day precision(CV)	< 2.17%	< 1.75%	2.4-10.4%	2.6-11.1%
Inter-day precision(CV)	< 9.52%	< 2.27%	1.7-17.6%	4-12.1%
Linearity	0.001-100 mg/L	0.001-100 mg/L	10^-5^-0.02 mg/L	1-960 mg/L
Detection time	1 hour	1.5 hours	2-6 hours	40 minutes

^a^Data are from the instruction of CRP detection reagent from Beckman Coulter

In addition to its high accuracy and sensitivity, the QL-MI method allows for high-throughput detection and automation. For the proposed hs-CRP assay, all of the operating procedures, including sample introduction, incubation, vortexing, washing, and fluorescence measurement, can be completed automatically by either a microplate reader or autoanalyzer. In addition, the 96-well microplate format is capable of simultaneously determining more than 90 samples in 1.5 h. This analytical time could even be shortened to < 1 h in a one-step format, making the entire process much faster than existing mass spectroscopy-based assays. Our future research is aimed at further improvements in throughput and standardization of the entire analytical process.

## Conclusions

A rapid and sensitive hs-CRP assay for the high-throughput determination of trace serum hs-CRP was developed by combining commercially available microplates and quantum dots labeling. The ability to read out the fluorescence signals conveniently using a microplate reader makes the proposed immunoassay an attractive candidate for large-scale CRP determination in various clinical laboratories even without the use of an autoanalyzer. This assay also provides the prospect for the high-throughput detection of other biomarkers by coating the microplate with specific monoclonal antibodies and by simply scaling up the 96-well microplate to a 384-well or higher microplate formats.

## Abbreviations

BSA: Bovine serum albumin; CV: Coefficients of variation; ELISA: Enzyme-linked immunosorbent assay; FI: Fluorescence intensity; hs-CRP: High-sensitivity c-reactive protein; hs-ELISA: High-sensitivity ELISA; mAbs: Monoclonal antibodies; PBS: Phosphate buffered saline; QD: Quantum dot; QL-MI: Quantum dot-labeled microplate immunoassay; SD: Standard deviation.

## Competing interests

The authors declare that they have no competing interests.

## Authors' contributions

YL have made substantial contributions to conception and design, data acquisition, analysis and data interpretation and are involved in drafting and revising the manuscript. WF has made substantial contributions to conception and design. BZ, MC, TJ, DZ, JH have made substantial contributions to data acquisition, analysis and data interpretation. Moreover, each author has taken public responsibility for appropriate portions of the content. All authors read and approved the final manuscript.
